# Alphaherpesvirus in Pets and Livestock

**DOI:** 10.3390/microorganisms13010082

**Published:** 2025-01-04

**Authors:** Shu-Hui Duan, Ze-Min Li, Xue-Jie Yu, Dan Li

**Affiliations:** 1 State Key Laboratory of Virology, School of Public Health, Wuhan University, Wuhan 430071, China; duansh@whu.edu.cn (S.-H.D.); 15234579641@163.com (Z.-M.L.); 2 Hubei Provincial Center for Disease Control and Prevention, Institute for Infectious Disease Prevention and Control, Wuhan 430079, China

**Keywords:** herpesviruses, alphaherpesviruses, pets, livestock

## Abstract

Herpesviruses are a group of DNA viruses capable of infecting multiple mammalian species, including humans. This review primarily summarizes four common alphaherpesviruses found in pets and livestock (feline, swine, canine, and bovine) in aspects such as epidemiology, immune evasion, and latency and reactivation. Despite the fact that they primarily infect specific hosts, these viruses have the potential for cross-species transmission due to genetic mutations and/or recombination events. During infection, herpesviruses not only stimulate innate immune responses in host cells but also interfere with signaling pathways through specific proteins to achieve immune evasion. These viruses can remain latent within the host for extended periods and reactivate under certain conditions to trigger disease recurrence. They not only affect the health of animals and cause economic losses but may also pose a potential threat to humans under certain circumstances. This review deepens our understanding of the biological characteristics of these animal alphaherpesviruses and provides an important scientific basis for the prevention and control of related diseases.

## 1. Introduction

The order *Herpesvirales* includes numerous viruses with a large double-stranded DNA genome that can potentially infect humans and almost all animal species [1]. The family *Herpesviridae* is divided into the three subfamilies of *Alpha-*, *Beta-*, and *Gammaherpesvirinae* based on their biological properties and morphology [2]. Currently, the International Committee on Taxonomy of Viruses (ICTV) classifies the *Alphaherpesvirinae* subfamily into five genera containing 49 species [3]. Alphaherpesviruses, including varicella-zoster virus (VZV) [4,5,6,7], herpes simplex virus types 1 and 2 (HSV-1 and HSV-2, respectively) [8,9,10,11], bovine herpesvirus 1 (BoHV-1) [12,13], and pseudorabies virus (PRV) [14,15,16], are prevalent in a wide range of host species and are characterized by their rapid replication process. The complete virion of alphaherpesvirus consists of a double-stranded DNA core, an icosahedral capsid, a tegument, and an envelope. The capsid, consisting of 150 hexagons and 12 pentagons, interacts with multiple viral and host proteins during the infection process. The nucleocapsid is embedded in a protein layer known as the tegument, which is further surrounded by a lipid bilayer envelope derived from the host cell membrane. The viral envelope contains multiple proteins including various glycoproteins with different functions. For example, glycoprotein D (gD) is primarily responsible for receptor recognition and initial attachment to host cells. Other glycoproteins, such as glycoprotein B (gB) and glycoprotein E (gE), play crucial roles in viral replication and cell-to-cell spread [17].

Alphaherpesviruses can cause a range of diseases, ranging from skin blisters to potentially life-threatening diseases: encephalitis, meningitis abortions, cancers, or postherpetic neuralgia [18]. These viruses initially infect the skin or mucosal epithelium and subsequently gain access to neuronal axons, traveling retrogradely to the neuron cell bodies in ganglia. Within the ganglia, the herpesviruses establish a state of lifelong latency, characterized by the dormancy of their pathogenic potential [19]. Due to their unique ability to enter a latent state, these viruses can persist as subclinical infections without the development of clinical signs. They can subsequently reactivate under specific conditions. Reactivation involves the transcriptional activation of viral genomes, followed by the synthesis of viral proteins and the assembly of new virions. It can lead to the re-emergence of clinical signs and enhances the likelihood of transmission to new hosts.

In hosts, particularly pets and livestock that share a living space with humans and engage in prolonged close contact, the possibility of the cross-species transmission of viruses is increased. It is reported that at least one of the most significant diseases affecting domestic animals is generally caused by herpesvirus. Some of these viruses have the potential to cause widespread outbreaks and may be transmitted between distantly related species [20]. This makes it important to pay more attention to alphaherpesviruses in these animals. Given the potential risks and the need for a better understanding of these viruses, this review aims to summarize the etiology, epidemiology, pathogenesis mechanisms, and antiviral treatment of four common alphaherpesviruses in domestic animals.

## 2. Felid Alphaherpesvirus 1

Feline upper respiratory tract disease (URTD) is a common disease, which has a great impact on cats worldwide, especially on cats living in communities or in close contact with other cats [21,22,23]. This disease is multifactorial, and several pathogens are involved; felid alphaherpesvirus 1 (FHV-1) is considered to play an important role in this disease.

According to the latest ICTV guidance, felid alphaherpesvirus 1 belongs to the genus *Varicellovirus* within the subfamily *Alphaherpesvirinae* of the family *Orthoherpesviridae*. The whole genome of FHV-1 is approximately 135 kb in length, consisting of unique long (UL) and unique short (US) gene sequences. It was first isolated in 1957 from a cat exhibiting respiratory signs [24]. Domestic cats are the primary hosts of FHV-1, and the cross-species transmission of FHV-1 naturally occurs more frequently in closely related host species. FHV-1 has also been isolated from various wild felid species, including captive, semi-captive, and free-roaming cheetahs [25,26,27]. Most cheetahs experience mild disease similar to that of domestic cats, while a minority develop severe ocular, upper respiratory tract disease, and dermatologic clinical signs [26,27]. Corneal ulcers and/or keratitis, as well as skin lesions, are more common in cheetahs with severe disease [28]. Additionally, FHV-1 has been detected in tigers, snow leopards, and chinchillas (Table 1) [29,30,31]. The primary transmission route of FHV-1 is through direct contact with infected cats. It can also be transmitted indirectly among susceptible cats via contaminated items such as food, water, and cages [32]. As with infection by other herpesviruses, FHV-1 can infect neurons, making the animal a lifelong latent carrier. Episodes of reactivation and dissemination can occur due to stress factors [25].

The clinical signs of URTD can be similar to those caused by various pathogens, including sneezing, nasal discharge, coughing, and conjunctivitis. FHV-1 is known to cause both upper respiratory and ocular signs. Corneal dendritic ulcers are a typical sign and are strongly indicative of FHV-1 infection [33]. It has been confirmed that different viral isolates can cause varying degrees of disease severity in different hosts, such as herpes simplex virus types 1 and 2. However, despite individual exceptions, the clinical signs observed in feline hosts are unlikely to be primarily associated with genomic variants of FHV-1 [34,35,36]. A previous global study on FHV-1 isolates revealed that the overall interstrain genetic distance among all available isolates was 0.093% [36]. Deng et al. also confirmed high sequence homology among three isolated FHV strains (FHV-A1, FHV-C8, and FHV-D3). Notably, the FHV-A1 isolate showed the highest virulence, making it a potential candidate for vaccine development [37]. These three strains showed a high identity with FHV-1 isolates from domestic cats as well as globally isolated FHV-1 viruses, consistent with earlier findings [31].

**Table 1:** Summary of clinical signs and natural hosts of FHV-1, PRV, CaHV-1, and BoHV-1.

Viruses	Clinical Signs	Natural Hosts (Host Range)	References
Felid alphaherpesvirus 1 (FHV-1)	Sneezing, nasal discharge, coughing, ocular disease, and conjunctivitis	Cats (cheetahs, tigers, snow leopards, and chinchillas)	[21,22,23,24,25,26,27,28,29,30,31]
Pseudorabies virus (PRV)	Temporary temperature elevation, respiratory signs, reproductive disorders, and occasional neurological signs	Wild boars and domestic pigs (cattle, sheep, goats, dogs, cats, foxes, minks, wolves, bears, raccoons, horses, chickens, and humans)	[15,38,39,40,41]
Canine herpesvirus 1 (CaHV-1)	Ocular, respiratory, and reproductive disorders	Canines	[42,43]
Bovine herpesvirus 1 (BoHV-1)	Respiratory diseases and reproductive failure	Cattle (goats, sheep, and water buffaloes)	[44,45,46,47]

The reported prevalence of FHV-1 is extremely variable (ranging from 2.6% to 63%), possibly due to the various sampling sites. The virus is commonly found in nasal, ocular, and oral samples, with a higher positive rate in nasal secretion compared to the oropharynx [33,48]. It has been noted that there is a significant association between FHV-1 infection and the occurrence of nasal discharge and sneezing, which are initial signs of upper respiratory tract involvement [22]. Additionally, testing for FHV-1 in non-vaccinated household cats has showed no significant correlation between gender and outdoor activity and FHV-1 infections. The distribution of FHV-1 appeared to depend largely on the route of viral shedding and the timing of sample collection [23].

To investigate the response of the cat respiratory tract to FHV-1 infection and to better understand the molecular pathogenesis and immune reactions, various cell models have been developed [49,50]. Feline respiratory epithelial cells cultured at the air–liquid interface (ALI-FRECs) morphologically resembled the natural airways of cats and have been established to address questions about immunity to FHV-1. ALI-FRECs are capable of supporting infection with and the replication of FHV-1. Additionally, ALI-FRECs can modulate the transcriptional regulation of various immune genes in response to viral infection, providing a valuable model for studying host–pathogen interactions [49]. Lee et al. developed a novel cell culture model using immortalized feline respiratory epithelial cells to investigate the innate immune response against FHV-1. This model provides a valuable tool to study a broad spectrum of other upper respiratory pathogens in cats, including but not limited to FHV-1 [50].

FHV-1 enters cells via pH- and dynamin-dependent endocytosis by interacting with an unknown entry receptor. Additionally, caveolin-1 is involved in the process of viral entry [51]. Previous studies have shown that two microRNAs (miRNAs: miR-101 and miR-26a) play significant roles in FHV-1 infection [52,53]. During the viral infection process, the expression levels of these miRNAs are upregulated via a cGAS-dependent pathway. At the same time, both miRNAs can enhance type I interferon (IFN-I) antiviral signaling by targeting and inhibiting the expression of the suppressor of cytokine signaling 5 (SOCS5), leading to increased STAT1 phosphorylation and the suppression of viral replication. Conversely, the Ser/Thr kinase US3 of FHV-1 inhibits IFN induction effectively in a kinase-independent manner by binding to IRF3 and preventing its dimerization, thus achieving immune evasion [54].

In clinical practice, nucleoside analogs are commonly used as antiviral drugs to combat FHV-1 infections. However, these drugs operate through a singular mechanism and can easily lead to drug resistance. Recently, small-molecule drugs have been screened, and it has been found that Saikosaponin B2, Punicalin, and Punicalagin could effectively exert antiviral effects in the early stages of viral infection. These findings show promise in the potential use of these compounds as antiviral drugs against FHV-1 [55]. At the same time, for eye diseases caused by FHV-1, drugs such as Ganciclovir and Famciclovir are worth further research and optimization to improve efficacy and reduce adverse reactions (Table 2) [56,57,58]. Lewin et al. investigated the genomic mutations of FHV-1 in domestic cats with ocular surface disease under different treatment conditions, but no functional variants were detected in the study [59]. Vaccination is also crucial for reducing the incidence rate and mortality of cats infected with FHV-1. An analysis of neutralizing antibody titers before and after FHV-1 vaccination revealed inconsistent antibody responses among vaccinated cats. Specifically, the effectiveness of the vaccine appeared to be less satisfactory in older cats and in purebred cats [60]. However, antibody levels were significantly higher in older cats compared to younger ones [61]. In order to improve the safety of commercially live vaccines, a new recombinant FHV-1 with deletions in the TK/gI/gE genes has been constructed using CRISPR/Cas9-mediated homologous recombination, which may serve as a live vaccine candidate (Table 2) [62].

## 3. Pseudorabies Virus

Pseudorabies virus (PRV) is also known as Aujeszky’s disease virus or Suid alphaherpesvirus 1 [38]. It can establish latent infection in swine with neurotropic properties. The virus was first described in 1813 and was identified as the causative agent of pseudorabies or Aujeszky’s disease in 1902 by a Hungarian veterinarian [39]. Wild boars and domestic pigs serve as the primary natural hosts and the only known latent carriers of PRV. The virus has been detected in a wide range of animals, including livestock (such as cattle, sheep, and goats) and wild animals (such as bears, foxes, and raccoons) (Table 1) [40]. Additionally, PRV infection represents a potential threat to humans as evidenced by cases confirmed through advanced diagnostic techniques such as ELISA, PCR, and metagenomics next-generation sequencing [15]. Significantly, the PRV strain hSD-1/2019 was isolated from the cerebrospinal fluid of a patient, providing direct evidence for PRV’s capability to infect humans [41]. Infections in humans are characterized by high fever, neurological symptoms, and visual impairment. In addition, some survivors suffered severe sequelae, including vegetativeness, cognitive impairment, and memory loss [39]. According to investigations, all patients had a history of exposure, but there is not yet sufficient evidence to confirm direct transmission from pigs to humans. Currently, there are no reported cases of human–human transmission.

Porcine pseudorabies has persisted in China for a long time, seriously threatening the nation’s livestock industry. The earliest PRV case in China dates back to 1947, involving a domestic cat. Due to the lack of an available PRV vaccine, the virus spread widely among swine populations in China. In the 1970s, a live attenuated vaccine, based on the PRV Bartha-K61 strain, was introduced into China and provided ideal protection against the prevalent classical strains at the time. However, despite the usage of the Bartha-K61 vaccine, frequent outbreaks of pseudorabies have occurred in pig farms since late 2011, with new emerging PRV variants leading to high mortality rates in newborn piglets. Genomic sequencing revealed that these new strains clustered in a distinct branch from previous classical strains. These newly isolated viruses were thus referred to as PRV variant strains [14,100]. PRV can be divided into two genotypes worldwide. Genotype I strains are widely prevalent in Europe, America, and China, whereas Genotype II strains predominantly originate from Asian countries, primarily China [100]. Regarding genetic diversity, genotype I of PRV strains can be divided into six subtypes. Notably, subtype 1.6 includes Chinese isolates that exhibit a close genetic relationship with the Bartha-K61 strain. On the other hand, Genotype II can be divided into two subtypes. Subtype 2.1 comprises classical strains that were isolated in China during the 1990s, and subtype 2.2 mainly consists of variant strains isolated after 2011 [39].

In recent years, provinces in China including Yunnan, Guangxi, Henan, Hunan, Hebei, and Shandong have conducted investigations on the prevalence, genetic variations, and virulence of PRV [101,102,103,104,105,106,107,108,109]. ELISA results targeting the gE antibody, which is one of the most widely applied serological approaches, revealed positive rates from 16.69% to 52.70% in Yunnan, Shandong, Henan, Hebei, and Hunan, with a higher positive rate in sows [103,104,105,109]. The PRV seropositive rate might be associated with the geographical distribution of pig farms, farm size and category, seasonal variables, and the cross-regional transportation of livestock [102,106]. Moreover, these studies have shown that PRV variants are prevalent in Bartha-K61-vaccinated pigs.

Currently, the strains circulating in domestic pig populations include vaccine strains, classical strains (such as Ea, Fa, and SC), and variant strains. Among these, the variant strains may have evolved from classical PRV strains through point mutation and recombination events [110]. Natural mutation–selection and positive selection have contributed to the diversity changes in PRV strains of genotype II, particularly at specific amino acid sites in the gB, gE, and gC glycoproteins [39]. These variant strains are of the primary genotype affecting the development of the swine industry [108]. It has been confirmed that recombinant strains, such as XJ5, derived from recombination between the Bartha vaccine strain and wild strains, exhibit enhanced pathogenicity. Genetically, these strains, including XJ5, do not differ significantly from classical strains [111,112]. The FJ62 strain might be a variant isolated from domestic pigs following natural recombination between genotype I of wild boar PRV and genotype II of swine PRV in China [113]. Additionally, coinfections with PRV, porcine circovirus type 2, porcine reproductive and respiratory syndrome virus, and classical swine fever virus were observed in pig populations [109].

PRV infection proceeds through several stages, including viral attachment, entry, replication, extracellular trafficking, and viral egress. During these processes, the critical initial steps that enable the completion of the viral life cycle are attachment and entry [114]. The binding and entry of PRV into target cells results from the coordinated action of viral proteins (such as gC, gD, gL, gH, and gB) and cellular receptors (such as nectins), and this process also involves various cellular factors, such as IFN-induced transmembrane proteins, Cholesterol 25-hydroxylase, and so on [115,116,117].

Initially, PRV’s attachment to the cell surface is so weak that it cannot trigger viral entry. During this phase, PRV gC and gB interact with the heparan sulfate receptor on the cell membrane. Subsequently, gD binds firmly to the cellular receptor nectins [118]. In addition, gB and gH serve as the essential fusion proteins. Notably, PRV gB is not an autonomous fusogen but requires a fusion signal from its partner gH/L to facilitate membrane fusion [119,120]. Previous studies have shown that mutations in gB, gC, and gD can modify the immunogenicity and pathogenicity of PRV variants in a mouse model [121,122]. During the viral entry process, nectin-1 and nectin-2 serve as the main receptors and are known for their involvement in various alphaherpesvirus infections. PRV gD is capable of directly interacting with nectin-1 in both humans and swine. Moreover, the binding affinities of PRV gD for nectin-1 are similar in both species. It is plausible that human nectin-1 might be involved in the cross-species transmission of PRV from pigs to humans [118]. Furthermore, viral glycoproteins could interact with neuropilin-1 [123] and thrombospondin 3 [124] to facilitate viral attachment and entry.

Once the virus enters the host cell, the viral DNA genome is transported to the cell nucleus, which triggers the host antiviral immune response. The host can activate an antiviral innate immune response by recognizing pathogen-associated molecular patterns (PAMPs) through pattern recognition receptors (PRRs). One of the most effective strategies for combating viral infections is the innate immune response mediated by IFN-I. Following infection, viral DNA is recognized by the cytoplasmic DNA sensor cGAS, which promotes the production of the second messenger cGAMP. Then, cGAMP binds to STING located on the endoplasmic reticulum, and they translocate to the Golgi apparatus, where they recruit TBK1 and IRF3/7. The phosphorylated IRF3/7 is transported into the nucleus to activate the transcription of IFN-I genes. Subsequently, IFN-I activates the JAK-STAT pathway, initiating the transcription of multiple ISGs to engage in antiviral functions [16]. However, PRV regulates IFN function in multiple ways, including IFN production and IFN signaling pathways, to achieve immune evasion, thereby promoting viral replication. UL41 could suppress the activation of PRV-induced inflammatory cytokines and negatively regulated cGAS-STING-mediated antiviral activity by targeting IRF3, inhibiting the translocation and phosphorylation of IRF3 [125]. Similarly, UL13, UL24, UL38, UL41, UL42, US2, US3, EP0, and gE could also antagonize IFN-I-mediated innate immune responses [125,126,127,128,129,130,131].

**Figure 1:**
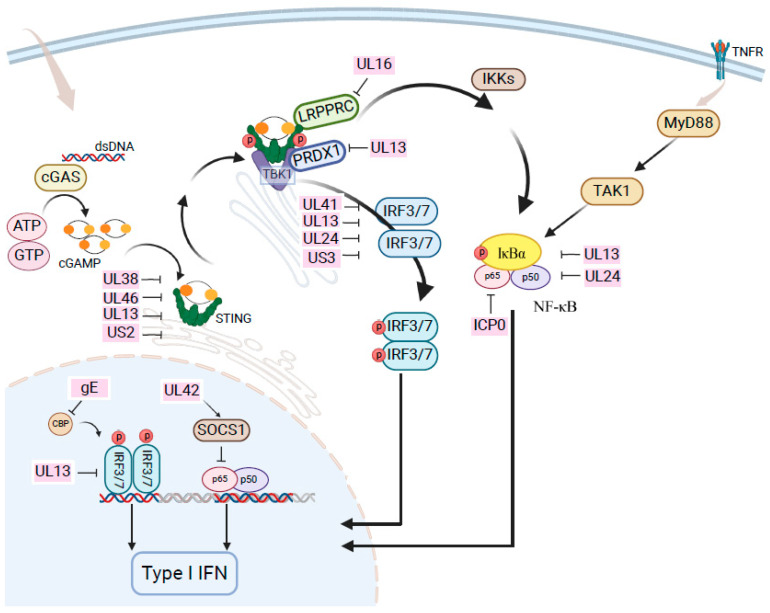
Pseudorabies virus regulates IFN-mediated innate immune responses by encoding multiple proteins.

UL24 and UL42 could also inhibit nuclear factor-κB (NF-κB) activation [132,133]. NF-κB serves as a critical regulator that, upon stimulation, transitions into an active state following the degradation of IκBα and then moves into the nucleus to initiate the transcription of various genes [16,134]. In one study, the PRV-encoded immediate early protein ICP0 inhibited NF-κB activation by interacting with the p65 subunit, promoting its degradation, and limiting its phosphorylation, thereby reducing the expression of inflammatory cytokines interleukin-6 (IL-6) and interleukin-8 (IL-8) [135]. PRV has been shown to manipulate the NF-κB signaling pathway in a unique manner, resulting in the activation of NF-κB but preventing it from carrying out its normal pro-inflammatory functions (Figure 1) [136,137].

Newly assembled herpesvirus nucleocapsids reach the cytoplasm for final virion maturation by vesicle-mediated nucleocytoplasmic transport across the intact nuclear envelope. The process begins with the budding of virions into primary enveloped particles at the inner nuclear membrane, within the perinuclear space. These particles fuse with the outer nuclear membrane to enter the cytoplasm [138]. The nuclear egress complex (NEC) is composed of two conserved viral proteins, designated pUL34 and pUL31, which play essential roles in the egress of herpesvirus nucleocapsids from the nucleus. However, the involvement of additional auxiliary steps or components in the complete transport process remains to be explored [139].

To enhance the prevention and control of pseudorabies, significant efforts have been made to develop effective strategies against PRV infection, including the development of vaccines and novel antiviral drugs (Table 2). The initial live gene-modified vaccines (the attenuated Bartha-K61 strain and the PRV Bucharest strain) are used widely in pig herds and have effectively controlled pseudorabies worldwide [140]. Since 2011, outbreaks of pseudorabies caused by emerging PRV variants have occurred in pig herds in China, indicating that classical attenuated vaccines like the Bartha-K61 strain cannot provide complete protection. In response, researchers have developed gene-modified vaccines based on novel virulent PRV strains, primarily by deleting genes such as gE, gI, and TK (Table 2). This review summarizes the latest advancements in vaccine improvement since 2022. A comprehensive overview of developments prior to 2022 has been written by Zheng et al. [140].

## 4. Canine Herpesvirus

Canine herpesvirus 1 (CaHV-1) causes canine infectious respiratory disease (CIRD), which is one of the most common diseases in dogs worldwide. The host range of CaHV-1 is limited to domestic and wild canids [42]. It can be transmitted through respiratory or genital routes. This virus is a significant cause of sudden death in neonates and is also associated with ocular, respiratory, and reproductive disorders [43]. Clinical signs vary depending on the age and immune status of the infected animal. Puppies under 2 weeks of age may present with a lethal, widespread necrotizing and hemorrhagic disease upon infection. In older puppies and adult dogs, the infection generally presents as an upper respiratory tract disease and may be associated with ocular disease (Table 1) [42].

Even though CaHV-1 has high seroprevalence among various canine populations with multiple associated clinical signs, only a small number of studies on the topic have been published. These studies mainly investigated the genomics and phylogeny of the virus, which was detected in kidney, spleen, nasal, and vaginal samples [141,142,143]. A genomic analysis of CaHV-1 isolates demonstrated that they exhibited a high similarity with each other. It suggested that CaHV-1 might have widespread transmission routes globally, not restricted by geographic boundaries [43]. In the vast majority of animals, antibodies were generated as a result of natural infection [144]. Significant risk factors for CaHV-1 infection included kennel size, some outdoor activities, kennel disinfection protocols, and mating [142]. In contrast to other alphaherpesviruses (FHV-1, PRV, and BoHV-1), there was no evidence of the subclinical ocular shedding of CaHV-1 in mature dogs [145]. This suggests that ocular shedding might not be a significant mode of transmission for CaHV-1 in mature dogs.

Therapeutic and preventive options for CaHV-1 infections are notably limited. Eurican Herpes 205, a gB subunit vaccine, is exclusively licensed in Europe and is particularly recommended for pregnant dams (Table 2). Currently, there are no commercial vaccines that provide lifelong protection against CaHV-1. Once animals develop acute signs, the available treatments focus on managing clinical signs and providing supportive care rather than curing viral infections.

## 5. Bovine Herpesvirus

Cattle serve as natural hosts for several herpesviruses including bovine herpesvirus 1 (BoHV-1), BoHV-2, BoHV-4, BoHV-5, and bovine lymphotropic herpesvirus (BLHV). Among these bovine herpesviruses, BoHV-1, BoHV-2, and BoHV-5 belong to the subfamily of *Alphaherpesvirinae*, while BoHV-4 and BLHV belong to the *Gammaherpesvirinae* subfamily. In this review, we primarily focus on BoHV-1, which is distributed worldwide and causes significant economic losses to the cattle industry. BoHV-1 mainly causes respiratory diseases (such as infectious bovine rhinotracheitis) and reproductive failure (such as infectious pustular vulvovaginitis/balanoposthitis) [44,45]. BoHV-1 has the potential to infect sheep and goat, which may play a role in inter-species transmission [46]. Fusco et al. has identified BoHV-1 genomes in aborted water buffaloes (Table 1) [47].

All BoHV-1 strains are divided into three subtypes: BoHV-1.1, BoHV-1.2a, and BoHV-1.2b. Most isolates of the BoHV-1.1 subtype are primarily obtained from cases of respiratory disease or abortion. BoHV-1.2 is isolated from genital organ lesions. Experimental evidence shows that different subtypes exhibit different clinical signs depending on the route of infection [146]. Among them, BoHV-1.2b seems to be the predominant strain of BoHV-1 in cattle in China and is a significant cause of bovine respiratory disease. At the same time, BoHV-1.2b isolates exhibit significant genetic differences [147,148]. Although animals infected with these three subtypes present varying clinical signs, these strains share common antigenic properties [149].

To identify potential risk factors in cattle, serological studies have been conducted. The results have shown that the seroprevalence of BoHV-1 is related to cattle age, herd size, the presence of goats in the herd, and artificial insemination. Additionally, infection with bovine viral diarrhea virus could make cattle more susceptible to BHV-1 infection [150,151,152,153]. Coinfection with bovine rabies virus and herpesvirus has been reported, but no significant association between these pathogens was identified [154]. In addition to different viruses causing coinfection, BoHV-1 modified live vaccines (MLVs) could also lead to disease in cattle. One study provided strong evidence that recombination between BoHV-1 MLV and wild-type viruses could occur naturally and caused disease [155]. After BoHV-1 infection, subsequent infection by *Mannheimia haemolytica* could significantly deteriorate the health status of animals. The interaction between *M. haemolytica* and BoHV-1 was dose- and time-sensitive. When a viral replication program had not yet been fully established, the proliferation of *M. haemolytica* could induce a pronounced suppressive effect on the virus [156].

Herpesviruses promote their replication and release in host cells following infection through multiple signaling pathways and molecular mechanisms. BoHV-1 encodes the UL49.5 protein, a small membrane protein that inhibits the function of the TAP complex, reducing the presentation of viral peptides. It helps the virus evade the host immune response [157]. Proline residues are essential for the UL49.5 protein to exert its binding and inhibition on the TAP complex [158]. Simultaneously, BoHV-1 infection can activate the epidermal growth factor receptor (EGFR) and its downstream effectors, such as phospholipase C-γ1 (PLC-γ1) and Akt kinase, to facilitate virus replication in Madin–Darby bovine kidney (MDBK) cells [159]. MDBK and CRIB cells are well-established and stably passaged cell lines, commonly used in laboratory research, including BoHV-1 studies [99]. In addition to the UL49.5 protein, UL41 and UL13 can also assist BoHV-1 in evading the host innate immune response by inhibiting the IFN signaling pathway, thereby facilitating viral replication [160,161]. Liu et al. reported that PLC-γ1 was able to regulate β-catenin signaling by affecting the subcellular localization of p-β-catenin [162]. They found that activated p-PLC-γ1 was located in the Golgi apparatus and showed a specific association with the viral protein gD. This suggested that PLC-γ1 signaling might facilitate the transport of progeny viruses out of the Golgi apparatus, which benefits virus productive infection [163].

Like other herpesviruses, BoHV-1 can also establish latent infections in neuronal cells, posing a continual threat due to its potential to reactivate under certain conditions. Following acute infection, BoHV-1 modulates innate immune responses in neuronal cells, such as the IFN signaling pathway and necroptosis, as well as inhibits the complement cascade. This modulation establishes a relative equilibrium between the virus and the neuronal cells, laying a foundation for viral latency within these cells [164,165]. As latency is established, the shedding of the infectious virus is not detected by virus isolation approaches. During the maintenance of latency, the only viral gene abundantly expressed is latency-related RNA, which encodes at least two miRNAs and a protein, namely, open reading frame 2 (ORF2) [166]. ORF2 RNA sequences and the Wnt/β-catenin signaling pathway can actively promote the maintenance of latency by partially impairing glucocorticoid receptor (GR)-mediated gene expression. The immediate early transcription unit 1 (IEtu1) promoter, containing two functional GR response elements (GREs), drives the expression of important viral transcriptional regulators (including bICP0 and bICP4) in the GR-mediated gene expression process [167]. Reactivation from latency periodically occurs. Under exposure to stressful stimuli, such as psychological stress and elevated corticosteroid levels, the expression of IEtu1 can be induced, promoting the reactivation of the virus from latency [168,169,170]. At the same time, in addition to the GR regulating gene expression, other transcription factors such as Krüppel-like transcription factor 15 (KLF15), KLF14, and the progesterone receptor also play a synergistic role, inducing reactivation from latency [171,172,173,174].

Many vaccines are available to control BoHV-1 infection in cattle (Table 2). To differentiate between vaccinated and truly infected cattle, gE gene-deleted marker vaccines are commonly used. Additionally, a commercial vaccine with deletions in both the gE and Tk genes plays a crucial role in disease control (Table 2) [175].

## 6. Comparative Analysis of Animal and Human Alphaherpesviruses

Alphaherpesviruses primarily infect skin and mucosal epithelial cells, causing localized inflammatory responses. CaHV-1 and FHV-1 are closely related viruses that share similar pathogeneses and induce similar ocular lesions to those caused by HSV-1 infections in patients, such as punctate and dendritic ulcerative keratitis [176,177], while FHV-1 not only affects respiratory epithelial cells but also specifically targets ocular and pulmonary cells, leading to conjunctivitis and respiratory tracts (Table 1) [178]. It has long been recognized that herpesvirus genomes contain several sites with tandem short sequence repeats (SSRs) or reiterations. The changes in length in SSRs have been associated with DNA binding site efficiency, transcription regulation, and protein interactions [39]. This genetic feature plays a crucial role in the viruses’ ability to adapt and infect different hosts. A genomic analysis of 26 FHV-1 isolates from different cats showed that they contain fewer SSRs compared to HSV-1 and HSV-2 [36]. PRV exhibits the highest overall SSR burden, with short repeats accounting for 18% of its genome. This proportion is roughly double that observed in HSV-1, and five to six times higher than in VZV [176].

Members of the Alphaherpesvirus subfamily commonly show a tropism for polarized cells with extensive cell-to-cell contact, enabling virion release at lateral cell junctions and the subsequent infection of adjacent cells. It has been confirmed that multiple alphaherpesviruses including RPV, BoHV-1, HSV-1, and HSV-2 can utilize nectin-1 as an efficient cellular receptor for cell entry. HSV-1 requires gD for cell entry and direct cell-to-cell spread, while PRV gD is involved in cell penetration but is dispensable in direct cell-to-cell spread [115]. Compared with HSV, the gD protein of PRV can only bind to nectin-1 at sites N77, I80, M85, and F129 [140]. Zhou et al. found that the gM homologs of PRV and other alphaherpesviruses (HSV-1 and VZV) shared the same mechanism for regulating apoptosis, enhancing viral replication and pathogenicity [179]. Different alphaherpesviruses and different host cells are involved in distinct endocytosis processes. For example, HSV-1 can enter human oligodendrocytic cells through clathrin-mediated endocytosis, while PRV can enter Hela cells via macropinocytosis. Cells internalize the virus–receptor complex on the cell membrane by activating downstream signaling pathways to facilitate viral entry, with different viruses triggering distinct downstream signaling pathways. The PI3K-Akt signaling pathway is triggered by HSV-1 and regulates viral entry. In one study, the BoHV-1 gD protein first interacted with nectin-1 to trigger the PI3K-Akt-NF-κB and Ras-p38 MAPK signaling pathways, and then these two signaling pathways promoted BoHV-1 to enter MDBK cells through clathrin-mediated and caveolin-mediated endocytosis [180]. Similarly to BoHV-1, PRV entered PK15 porcine cells through clathrin-mediated endocytosis [181]. The FHV-1 infection complex affected the PI3K/Akt/mTOR signaling pathway. It has been shown that an increase in Akt phosphorylation was observed during early viral infection, suggesting that the pathway might be activated and involved in the process of viral entry into cells [182].

## 7. Conclusions

Alphaherpesviruses are known to cause different diseases in humans and animals. Transmission primarily occurs through direct contact with mucosal secretions from infected individuals. Additionally, indirect transmission may happen through contact with contaminated objects. In some cases, vertical transmission can also occur. These viruses possess a complex genome structure that can encode numerous proteins, participating in viral replication, assembly, and interactions with host cells. Viral entry depends on specific glycoproteins such as gB, which undergo conformational changes upon activation to promote the fusion of the viral envelope with the host cell membrane.

Herpesviruses establish lifelong latent infection and recurrent lytic infections in both humans and animals. In order to evade the immune responses of their hosts, they have evolved a variety of strategies. To maintain this delicate balance between latency and reactivation, alphaherpesviruses employ multiple mechanisms to modulate host immunity. An in-depth understanding of the latency and reactivation mechanisms of alphaherpesviruses is essential not only for an understanding of viral pathogenesis and host–virus interactions, but also for identifying potential targets for therapeutic intervention.

## Figures and Tables

**Table 2:** Vaccines and antiviral compounds for FHV-1, PRV, CaHV-1, and BoHV-1.

Viruses	Vaccine	Antiviral Drugs	Other Antiviral Compounds
FHV-1	Fel-O-Vax^®^ PCT vaccine [37]	Idoxuridine, Vidarabine, Trifluridine, and Cidofovir [63]	Lysine, IFNs, Lambda-carrageenan [63]
	Modified live or inactivated FVRCP vaccine [64]	Acyclovir and Valacyclovir [63]	Leflunomide, Lactoferrin, Small interfering RNAs [63]
	PUREVAX^®^ RCPCh FeLV [65]	Ganciclovir and Valganciclovir [63]	Probiotics [63]
	WH2020-ΔTK/gI/gE [62]	Penciclovir and Famciclovir [63]	Sinefungin [66]
		Bromovinyldeoxyuridine, Adefovir, PMEDAP, and HPMPA [63]	Ciclosporin [67]
		Raltegravir [68,69]	
		Saikosaponin B2, Punicalin, and Punicalagin [55]	
PRV	Attenuated Bartha-K61 strain and PRV Bucharest strain	Brincidofovir [70]	*Morus alba* L. extract [71]
	rPRV-2Cap/3Cap and rPRV-2Cap/3Cap/IL4 [72]		Baicalin [73]
	ZJ01-ΔgI/gE/TK/UL21 [74]		*Rehmmannia glutinosa* polysaccharide [75]
	rHN1201^TK−/gE−/gI−/11k−/28k−^ [76]		Polyethylenimine [77]
	PRV^ΔTK&gE-US3deop−1^ [78]		Salvianolic acid A [79]
	PRV^ΔTK&PK&gE-AH02^ [80]		Dihydromyricetin [81]
	PRV Δ gE/TK/US3 [82]		*Bacillus subtilis* [83]
	PRV XJ Δ gI/gE/TK [84]		Luteolin [85]
	SD-2017Δ gE/gI/TK [86]		*Hippophae rhamnoides* polysaccharides [87]
	LSgD nanoparticle vaccine [88]		Glycyrrhiza polysaccharide [89]
	rPRV-XJ-ΔTK/gE/gI-VP3 [90]		Myricetin [91]
	PRV GX-ΔTK/IES [92]		Resveratrol [93]
	gB + gD + GM-CSF-based subunit vaccine [94]		
	PRV-gD-based mRNA vaccine [95]		
CaHV-1	Eurican Herpes 205^®^ [42]	Ganciclovir [96]	
BoHV-1	BHV-gI gE-/eGFP+ mutant [97]		
	gE-live, gE-killed, gG-killed, gC-live, gD-subunit, gB-subunit, and gD-replication-incompetent [98]		
	ΔgE/TK IBR vaccine [99]		
	BoHV-1 gG−/tk− mutant [99]		
	Modified live virus vaccines [99]		
	Inactivated vaccines [99]		
	gD-subunit and gB-subunit [99]		
